# Unlocking health insights: exploring intention to adopt district health information systems in Bahir Dar City, northwest Ethiopia

**DOI:** 10.3389/fdgth.2025.1449510

**Published:** 2025-02-25

**Authors:** Habtamu Alganeh Guadie, Amarech Kindie, Hunegnaw Almaw Derseh, Desta Debalkie Atnafu

**Affiliations:** ^1^ Department of Health System Management and Health Economics, School of Public Health, College of Medicine and Health Sciences, Bahir Dar University, Bahir Dar, Ethiopia; ^2^ Amhara Regional Health Bureau, Bahir Dar, Ethiopia; ^3^ Department of Nutrition and Dietetics, School of Public Health, College of Medicine and Health Sciences, Bahir Dar University, Bahir Dar, Ethiopia

**Keywords:** intention, district health information system, primary health, health center, Ethiopia

## Abstract

**Background:**

District Health Information System version 2 (DHIS2) is an open-source platform designed for data collection, processing, analysis, and visualization within healthcare systems. However, there is limited empirical evidence regarding health professionals’ intentions to use district health information systems. Understanding the factors influencing health workers’ intention to utilize DHIS2 is crucial for ensuring successful implementation and sustained usage. This study aimed to assess the intention to use DHIS2 and identify associated factors among health professionals in health centers of Bahir Dar Metropolitan City, Ethiopia, in 2022.

**Methods:**

An institutional-based cross-sectional study was conducted involving 368 randomly selected health professionals from health centers in Bahir Dar City, Ethiopia, between 24 May and 24 June 2022. Data were collected using a structured, self-administered questionnaire. Completed questionnaires were entered and coded in EpiData version 4.6 and exported to SPSS version 25 for cleaning and statistical analysis. Descriptive statistics and bivariate and multivariable logistic regression analyses were performed. Model fitness was assessed using the Hosmer–Lemeshow goodness-of-fit test, with statistical significance set at a *p*-value < 0.05 and a 95% confidence interval.

**Results:**

A total of 342 participants completed the study, resulting in a response rate of 92.9%. The sample included 176 (51.5%) women, of whom 147 (43%) were nurses. Nearly two-thirds (65.2%) of health professionals expressed an intention to use the DHIS2 system. The intention to use DHIS2 was significantly associated with factors including attitude, computer skills, perceived utility, and perceived ease of use.

**Conclusion:**

The findings indicate that attitude, perceived utility, perceived ease of use, and computer skills significantly influence the intention to utilize DHIS2. Therefore, it is imperative to implement targeted interventions before system rollout, including practice-based training, fostering positive attitudes, and enhancing knowledge of the system's usability and functionality to improve the adoption of the district health information system.

## Background

The World Health Organization (WHO) defines a healthcare information system as one that integrates information collection, handling, examination, reporting, and utilization to increase the effectiveness and utility of healthcare services by enhancing oversight across the board for healthcare practices. The system aims to integrate various healthcare data for improved management, analysis, and decision-making across all levels of a healthcare system (1).

District Health Information System version 2 (DHIS2) is an open-source, flexible offline and online program that gathers, verifies, analyzes, and displays personalized data in order to organize and integrate patient-specific statistics information with integrated healthcare data. DHIS2's overarching objective is to produce high-quality health information that can be utilized and shared with the right people in the right situations in the right ways (2, 3).

To support efficient policy formulation, management, planning, budgeting, implementation, monitoring, and evaluation of health services and program interventions in the health sector, data from the DHIS2 is gathered, analyzed, and reported (4, 5).

The majority of health professionals in developing countries who want to use DHIS2 encounter challenges such as inadequate ICT infrastructure as well as a lack of computer proficiency among health professionals, expertise, and training (6, 7).

Evidence showed that attitude, perceived usefulness, and perceived usability all have a direct impact on health professionals’ intentions to utilize HMIS/DHIS. Similarly, users’ perceptions of utilizing DHIS2 were significantly impacted by inadequate human resources, bad Internet connectivity, and a poor culture of DHIS2 usage (8–14).

The adoption of DHIS2 in Kenya was a positive development. Nonetheless, there is strong evidence that health professionals are reluctant to employ health technology, which adds to the health sector's delayed adoption and use of DHIS2 (15).

The study's findings indicate that online reporting was challenging for Uganda's lower-level HMIS users. The DHIS2 platform often slows down during peak reporting periods due to several users overloading the web bandwidth. Furthermore, reports containing errors and missing data are inaccurate, untrustworthy, and difficult to submit. Users have a difficult time with this experience (16).

Furthermore, in Tanzania, vertical program data reporting systems frequently duplicate or overlap with the national main HMIS data, making it difficult to determine which data to use and increasing the burden on health providers who must record multiple sets of HMIS data (17).

Ethiopia began customizing and adopting DHIS2 software in 2017 (13, 18). All previously deployed and current systems have been put into place using a very costly and ineffective trial-and-error method. Therefore, the adoption and spread of HIS technology in Ethiopia are still in their early stages.

However, evidence is lacking in Ethiopia regarding the intention to use DHIS2. This study aimed to assess health professionals’ intention to use DHIS2 and its associated factors in the Ethiopian healthcare context. Understanding this is crucial for identifying critical factors that affect the adoption of digital health systems, which are essential for improving health data management and ultimately enhancing healthcare delivery.

## Methods

### Study design, period, and setting

A cross-sectional study design was conducted among health professionals in the health centers of Bahir Dar Metropolitan City between 24 May and 24 June 2022. The study was conducted at Bahir Dar Metropolitan City health centers. Bahir Dar is the capital city of Amhara National Regional State. It is located in the northwest highlands of Ethiopia. There are 19 health centers in Bahir Dar Metropolitan City, including Han HC, B/dar HC, Shimbt HC, Abay HC, Shumabo HC, D/minilik HC, Meshenti HC, Zege HC, Zenzelma HC, T/abay HC, F/wega HC, Leta HC, Sekelegna HC, Dek HC, Robit HC, Kinbaba HC, Andasa HC, Yinesa HC, and Wenjeta HC. Approximately 706 health professionals work in Bahir Dar Metropolitan City Health Centers.

### Source and study population

All health professionals who worked at health centers in Bahir Dar City were the source population for our study, whereas all health professionals who worked at selected health centers in Bahir Dar City and who were available during the data collection period were our study populations.

### Inclusion and exclusion criteria

The study included medical professionals who worked at health facilities. Health professionals who were not working permanently and who in any way missed work during the data collecting period were not included in our study.

### Sample size determination and sampling procedures

In order to address the main objectives, the sample size was calculated using a single population proportion formula, the 5% margin of error, the 95% confidence interval, and considering the 15% non-response rate for intention to use as *P* = 39.8% (19).

$$\begin{array}{c} n=\frac{\left(Za/2)2\;P(1-P\right)}{d^{2}}\\ n=369 \end{array}$$

Since the total number of source population during the study period was less than 10,000, a correction formula was also used.

$$nf=\;\;\;\;\;\;\frac{n_{o}}{1+\frac{n_{o}}{N}}\;\;=\;\frac{369}{1+\frac{\;369}{504}}\;=213$$

By considering the non-response rate of 15%, the sample size was 245. A multi-stage sampling technique was used. Then, we considered a design effect of 1.5 and the final sample size was 368. Hence, this sample size ensures that the responses of study participants are sufficiently reliable to generalize. From the 19 health centers, 12 were selected using a simple random sampling technique; the 12 randomly selected health centers contained 504 health professionals. Finally, the health professional was selected using a computer-generated simple random sampling technique.

### Study variables

The outcome variable was the intention of health professionals of nurses about DHIS2. Factors such as age, sex, education status, work experience, profession, supportive supervision, training on DHIS2, financial and material support, ICT infrastructure in organizations, perceived usefulness, perceived ease to use, attitude, and computer skills were independent variables in this study. The above variables were adapted from various studies (19–21).

### Operational definitions

#### Intention to use DHIS2

This refers to the measured likelihood or willingness of healthcare professionals to adopt and utilize the DHIS2 software for health information management. It was measured using three Likert-scale questions, and study participants who scored above the mean from the three Likert scale questions were considered as having good intention to use (19).

### Computer skills

Computer literacy refers to the ability of an individual to effectively use computer systems and software to accomplish tasks. This includes not only basic skills but also the capability to understand and apply various technologies in everyday activities. In this study, we used a 5-point Likert scale ranging from strongly disagree to strongly agree. Study participants who scored above the mean were considered to have computer skills, while those who scored below the mean were considered not to have computer skills (20).

#### Attitude

Study participants were asked a series of six questions about their feelings regarding DHIS2. Each question utilized a 5-point Likert scale ranging from “strongly disagree” to “strongly agree.” Finally, a mean score cutoff point was used to classify attitudes: participants with a score less than or equal to the mean score were categorized as having an “unfavorable attitude,” while those with a score greater than the mean score were classified as having a “favorable attitude” (21).

#### Perceived usefulness

Perceived usefulness refers to the extent to which a healthcare professional believes that using the health information system would enhance and improve their performance. It had 5 points on a Likert scale component, ranging from strongly disagree to strongly agree. Study participants who scored above the mean were considered as perceived to be useful and those who scored less than the mean were considered as perceived to be not useful (20).

#### Perceived ease of use

Perceived ease of use refers to the level to which a healthcare professional believes that they will be free from physical and mental effort to use health information systems. It had 4 points on a Likert scale component, ranging from strongly disagree to strongly agree. Study participants who scored above the mean were considered as perceived to be easy and those who scored less than the mean were considered as perceived to not be easy (20).

### Data collection tool and procedure

A standardized questionnaire that was customized for this study and adapted from related literature was used to gather data. Demographic data, an intention to utilize DHIS2, perceptions of utility and usability, attitudes, and computer skills were all included in the questionnaire. The tool was adapted after reviewing different literature (19–21). A self-administered questionnaire with closed-ended questions was used to collect the data. In addition, questions with a 5-point Likert scale were used to assess each item (check Supplementary File). The items’ attitude, perceived utility, perceived ease of use, and intention to use were all evaluated. Structured questionnaires facilitate data analysis since they record data quickly and accurately. Three people were hired to gather the data, and a public health officer was hired to oversee the entire data collection procedure.

### Data quality assurance

Five data collectors and two supervisors received 2 days of training on the objective of the study, relevance of the study, confidentiality of data, respondents right, informed consent, and data collection procedure during data collection. We used well-designed, pre-tested questionnaires. The pre-test was conducted among 37 health professionals (10% of the total sample size) at a health center, which was similar to our study setting.

Based on the results of the pre-test, the questionnaires were examined, reviewed, and checked for completeness and content validity. Cronbach's alpha was calculated to determine reliability; the overall Cronbach's alpha value for perception-related questioners was 0.854.

### Data processing and analysis

Completed questionnaires were entered and coded using EpiData version 3.1, and the data were then exported to SPSS version 25 for further cleaning and statistical analysis. Frequency tables and descriptive summaries were employed to describe the study variables. A binary logistic regression model was applied to identify determinants of the intention to use DHIS2. Variables with a *p*-value <0.25 in the bivariable analysis were selected as candidates for the multivariable logistic regression analysis. The fitness of the multivariable model was evaluated using the Hosmer–Lemeshow test, with a resulting *p*-value of 0.318, indicating an acceptable model fit. The outputs of both bivariable and multivariable analyses were reported using crude odds ratios (COR) and adjusted odds ratios (AOR), along with their 95% confidence intervals. Variables that showed statistical significance (*p* < 0.05) in the multivariable analysis were identified as important determinants of the outcome variable. The study findings were presented comprehensively through tables, figures, frequencies, and narrative texts to ensure clarity and effective communication of the results.

## Results

### Sociodemographic characteristics

Out of 368 individuals, 342 responded, resulting in a response rate of 92.93%. Of the respondents, 175 (51.5%) were women. More than half of the respondents (*n* = 186, 53.7%) aged 30–39 (mean age 31.34 ± 5.5). More than one-third (*n* = 147, 43%) of the study participants were nurses. One-third (33.6%) of the study participants had less than 3 years of work experience (Table 1).

**Table 1:** Sociodemographic characteristics for health professionals in Bahir Dar Metropolitan City, north west Amhara, Ethiopia, 2022 (*n* = 342).

Socio demography characteristics		Frequency (%)
Sex	Male	166 (48.5)
	Female	176 (51.5)
Age	20–29	129 (37.7)
	30–39	183 (53.5)
	40–49	30 (8.8)
Marital status	Currently married	220 (64.3)
	Currently unmarried	122 (35.7)
Educational status	Diploma	130 (38)
	Bachelor’s degree	199 (58.2)
	MSC and above	13 (3.8)
Profession	Health officer	44 (12.9)
	Laboratory technician	32 (9.4)
	Pharmacist	45 (13.2)
	Nurse	147 (43)
	Midwife	48 (14)
	Others	26 (7.6)
Experience	1–3	115 (33.6)
	4–6	106 (31)
	7–9	53 (15.5)
	≥10	68 (19.9)

### Attitude and perceived easiness toward intention to use DHIS-2

Of the total respondents, 106 (31%) had a favorable attitude toward the intention to use DHIS2, a software platform designed for health information management. This indicates that a significant portion of the participants recognize the potential benefits of DHIS2 in improving data collection, reporting, and decision-making processes in healthcare settings. The overall perceived ease of use among the 342 respondents showed that 161 (47.1%) found DHIS2 easy to use, while 181 (52.9%) did not find it easy. More than half of the respondents felt that it was easy for them to learn how to use DHIS2, and 187 believed that they could become skilled at using it. In addition, 184 (53.8%) agreed and 23 (6.7%) strongly agreed that they were flexible when interacting with DHIS2.

### Perceived usefulness toward intention to use DHIS-2

Of the 342 respondents, 214 (62.6%) perceived DHIS2 to be useful. Among the respondents, 66 (19.3%) agreed that DHIS2 improved their effectiveness in healthcare delivery, while 186 (54.4%) strongly agreed. In addition, more than two-thirds of the respondents believed that DHIS2 increased their productivity.

### Computer skill toward intention to use DHIS-2

Of the overall respondents, 164 (48%) demonstrated computer skills, indicating that nearly half of the participants were proficient in using basic computer applications and navigating digital platforms. This level of computer literacy is essential for effectively utilizing software like DHIS2, which relies on users’ ability to manage and analyze health data.

### Organizational factors

Among the total of 342 respondents, 301 (88%) had no prior experience using DHIS2. In contrast, 48 (14%) of the respondents had attended training on DHIS2. This lack of experience may have an impact on their willingness to adopt the software, highlighting the importance of training programs to enhance users’ familiarity and confidence in utilizing DHIS2 for health information management (Table 2).

**Table 2:** Organizational determinants that affect intention to use DHIS2 among health professionals in Bahir Dar Metropolitan City, north west Amhara, Ethiopia, 2022.

Variables	Frequency (%)	
	Yes	No
Ever use of DHIS2	41 (12)	301 (88)
Person available to assist DHIS2 in your facility	163 (47.7)	179 (52.3)
Instructions available in your facility	30 (8.8)	312 (91.2)
Training programs available in your facility	34 (9.9)	308 (90.1)
Did you attend training on DHIS2?	48 (14)	294 (86)
How many days of training on DHIS2	3–5 days	39 (81.3)
	6–7 days	5 (10.4)
	8–10 days	1 (2.1)
	>10 days	3 (6.3)
In which form was the training taken	Only theoretical	16 (33.3)
	Both theoretical and practical	32 (66.7)
Necessary equipment available for implementation	123 (36)	219 (64)
A power supply is available	88 (71.5)	35 (28.5)
Telephone is available	62 (50.4)	61 (49.6)
Computer hardware and software available	81 (65.9)	42 (34.1)
Internet or any kind of network available	Yes 40 (32.5)	83 (67.5)
Internet access in your facilities	Yes 148 (43.3)	194 (56.7)
How would you rate the internet access	Very satisfied	13 (8.6)
	Satisfied	46 (30.5)
	Unsatisfied	63 (41.7)
	Very unsatisfied	29 (19.2)

### Intention to use DHIS2

**Figure 1:**
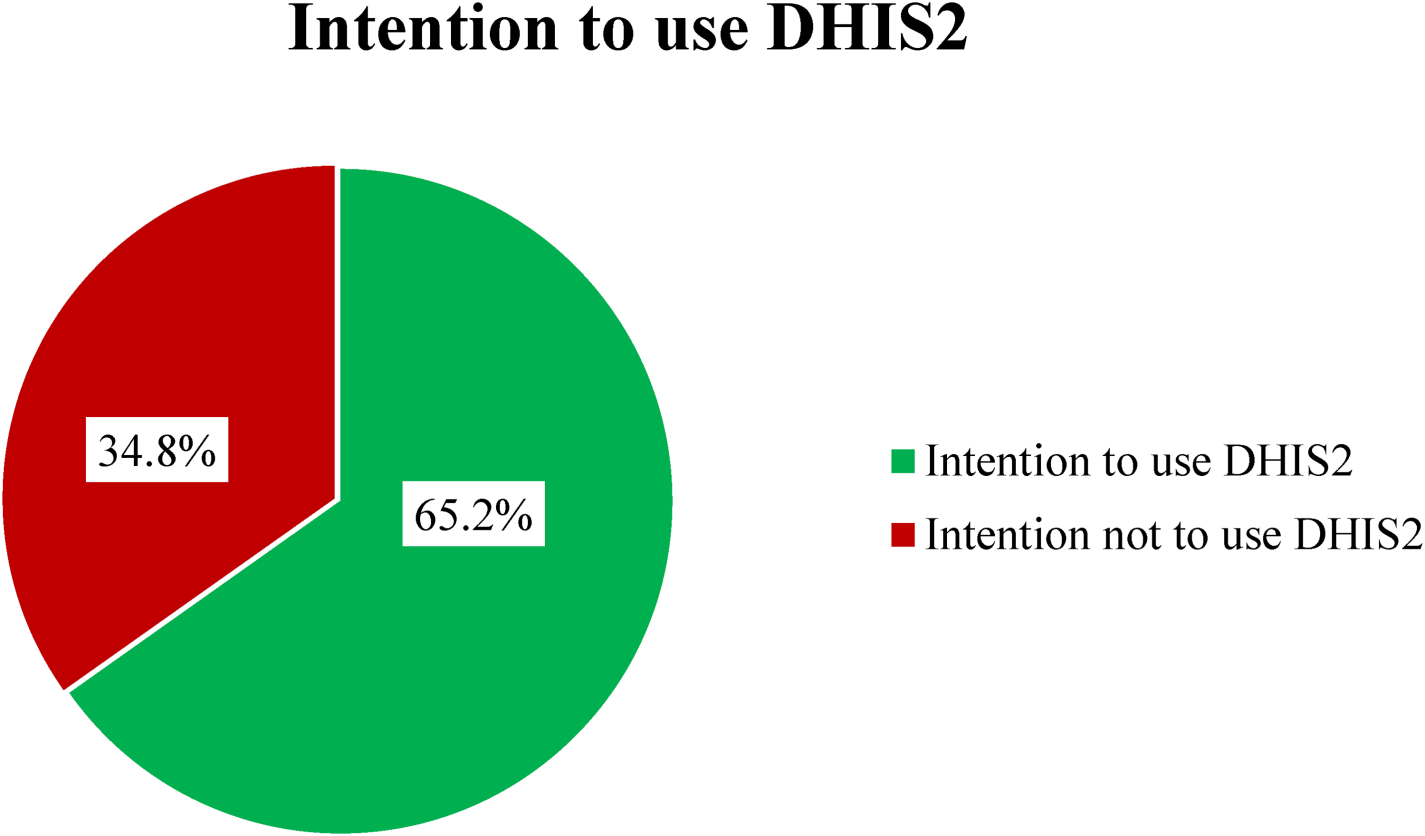
Intention to use DHIS2 among health professionals in Bahir Dar Metropolitan City, north west Amhara, Ethiopia, 2022.

In this study, 223 (65.2%) participants indicated an intention to use DHIS2 (95% CI = 60–70). This suggests that a significant majority of the respondents recognize the potential benefits of DHIS2 for improving health data management and reporting, which may lead to better decision-making in their respective healthcare environments (Figure 1).

### Factors associated with intention to use DHIS2

All variables with a *p*-value < 0.25 in the bivariate analysis were entered into the multivariable logistic regression to identify the associations between the dependent and independent variables.

Health professionals with an unfavorable attitude were 77% less likely to intend to use DHIS2 compared to their counterparts. Those with computer skills were 4.81 times more likely to express an intention to use DHIS2 than those without computer skills. In addition, health professionals who perceived DHIS2 as useful were 3.25 times more likely to intend to use it compared to those who perceived it as not useful. Conversely, health professionals who found DHIS2 difficult to use were 86% less likely to intend to use it compared to those who found it easy to use.

These findings highlight the critical role of attitudes, computer literacy, and perceived usability in influencing the intention to adopt DHIS2 among health professionals (Table 3).

**Table 3:** Multivariable logistic regression result on intention to use DHIS2, among health professionals in health centers of Bahir Dar Metropolitan City, north west Amhara, Ethiopia, 2022.

Variable	Intention		COR (95%) CI	AOR (95%) CI	*p*-Value
	Intention to use	Intention not to use			
Marital status					
Currently married	134	86	0.58 (0.36–0.94)	0.51 (0.16–1.57)	
Currently unmarried	89	33	1	1	
Attitude					
Unfavorable attitude	**132**	**104**	**0.21 (0.11–0.38)**	**0.23 (0.05–097)**	**0**.046*
Favorable attitude	91	15	1	1	
Did you attend training					
Yes	38	10	2.24 (1.07–4.67)	1.02 (0.18–5.77)	
No	109	185	1	1	
Computer access					
Yes	64	17	1.88 (0.82–4.34)	1.38 (0.48–4.01)	
No	28	14	1	1	
Internet access					
Yes	34	6	2.44 (0.91–6.55)	2.09 (0.62–702)	
No	58	25	1	1	
Computer skill					
Have computer skill	**144**	**34**	**4.56 (2.81–7.39)**	**4.81 (1.41–16.42)**	**0**.012*
Have not computer skill	79	85	1	1	
Perceived usefulness of DHIS2					
Perceived useful	**179**	**35**	**9.76 (5.84–16.33)**	**3.25 (1.05–10.10)**	**0**.041*
Perceived not useful	44	84	1	1	
Perceived easiness of DHIS2					
Perceived not easy	**79**	**102**	**0.09 (0.05–0.16)**	**0.04 (0.037–0.54)**	**0**.004*
Perceived easy	144	17	1	1	
Instruction availability					
Yes	24	6	2.27 (0.90–5.72)	7.84 (0.69–9.46)	
No	199	113	1	1	

1, reference; COR, crude odds ratio; AOR, adjusted odds ratio. The bold indicates the significance of variables.

* *p*-Value <0.05 shows a significant association.

## Discussion

This study revealed that nearly two-thirds (65.2%) of the respondents expressed a strong intention to use DHIS2 in health centers. This finding highlights the critical role of engaging healthcare professionals in the implementation of health information systems. Assessing the goals, perspectives, and needs of health professionals and end users is essential for ensuring the success and long-term sustainability of such systems. Actively involving stakeholders during the design and rollout phases can enhance usability, foster a sense of ownership, and ensure the system aligns with users’ practical requirements. Ultimately, this approach contributes to improved system adoption and the overall quality of healthcare delivery.

However, the results of this study are lower than those reported in studies conducted in Cameroon (22), Ghana (23), and Botswana (6). Possible explanations for this discrepancy include inadequate support for training, inconsistent Internet connectivity, unreliable electricity supply, and insufficient access to computers in health centers. These factors can significantly hinder the effective adoption of DHIS2, as they limit healthcare professionals’ ability to engage with the system and develop the necessary skills for its use. Addressing these barriers is essential to enhance the overall intention to use health information systems in similar contexts.

This study found a strong positive association between perceived usefulness and the intention to use DHIS2, with an AOR of 3.25 (*p* = 0.041). This suggests that individuals who perceive DHIS2 as a useful tool are 3.25 times more likely to intend to use it compared to those who do not share this perception. This finding may be explained by the fact that health professionals who recognize the practical benefits of DHIS2, such as improving efficiency, data management, or decision-making, are more motivated to adopt and integrate the system into their practice. Recognizing and addressing the factors that enhance perceived usefulness could therefore be a key strategy for promoting DHIS2 adoption. Previous studies (6, 23, 24) have similarly found that health professionals with favorable expectations of DHIS2 are more likely to intend to use the system than their counterparts.

Perceived ease of use exhibited a very strong positive association with the intention to use DHIS2, with an AOR of 25 (*p* = 0.004). This indicates that individuals who perceive DHIS2 as user-friendly are significantly more likely to intend to use the system. This relationship can be explained by the technology acceptance model (TAM), which posits that ease of use is a critical factor influencing the acceptance and adoption of technology. Health professionals who view DHIS2 as intuitive and straightforward to operate are more likely to adopt it, as lower usability barriers reduce resistance and increase engagement. These findings underscore the importance of designing health information systems that prioritize usability to enhance adoption rates and sustained utilization (6, 23, 24). Ease of use reduces the cognitive load associated with learning new technology, thereby facilitating its acceptance and integration into clinical settings.

The AOR of 4.81 (*p* = 0.012) indicates a strong positive association between computer skills and the intention to use DHIS2, suggesting that individuals with better computer skills are significantly more likely to intend to use the system. Proficiency in computer skills is a critical enabler for the effective utilization of health information systems such as DHIS2, as it facilitates system navigation, data entry, and interpretation. This study highlights the pivotal role of computer skills in shaping the intention to adopt DHIS2. The finding aligns with previous research conducted in Malaysia, which identified computer skills as a key determinant of healthcare technology adoption. This underscores the need for targeted training programs to enhance computer literacy among healthcare professionals, thereby promoting broader and more effective use of digital health technologies (25). Health professionals with strong computer abilities are more inclined to appreciate the value of computer-based systems in their work. Their proficiency with technology, combined with access to suitable computers, resources, training opportunities, and support from various stakeholders, likely increases their comfort in adopting digital tools compared to those lacking such support and capabilities. Thus, the relationship between skill level and positive perceptions may be partially attributed to the infrastructure and environment that fostered the development of both among certain practitioners. In addition, health professionals with computer knowledge can more effectively research, update, and access scientifically backed sources related to electronic health records and treatments.

According to this study, healthcare professionals’ attitudes toward DHIS2 positively influenced their intention to use the system. An AOR of 0.23, with a *p*-value of 0.046 for unfavorable attitudes, indicates that the reciprocal of this unfavorable attitude (1/0.23 = 4.35) suggests that for every one-unit increase in favorable attitude toward DHIS2, the odds of intending to use it are approximately 4.35 times higher compared to someone with an unfavorable attitude. These results align with a study conducted in Ghana, which demonstrates that attitudes toward technology have a strong statistically significant influence on healthcare professionals’ intention to use healthcare technology (23). A possible explanation for this finding is that health professionals with favorable attitudes are more likely to intend to use the system as a result of their beliefs, personal interests, and behaviors. Positive attitudes can facilitate openness to new technologies, making it easier for professionals to engage with and utilize systems like DHIS2 effectively. This study has certain limitations. First, a theoretical framework was not applied such as UTAUT or TAM. Second, the self-reported nature of the data introduces the potential for social desirability bias, where participants may have given responses they deemed more socially acceptable rather than completely accurate.

## Conclusions

This research provides valuable insights on intention to use and its associated factors toward the adoption of DHIS2 among health professionals in health centers in Bahir Dar City. The findings indicate that two-thirds of health professionals intend to use DHIS2, highlighting a significant potential for its implementation in improving health data management and decision-making.

Key factors identified in this study, such as attitudes, perceived benefits, computer skills, and the perceived ease of use, play a critical role in enhancing the adoption of DHIS2. Specifically, improving healthcare professionals’ attitudes toward the system, demonstrating its perceived benefits, and enhancing computer skills are essential strategies for facilitating its adoption. Ongoing training programs focusing on computer skills and the practical applications of DHIS2 will empower healthcare professionals to utilize the system effectively.

Furthermore, we recommend that future researchers should explore mixed methods to contextualize quantitative insights with qualitative depth to explore the primary challenges associated with the implementation of digital health systems in developing countries, particularly in Ethiopia. Understanding these challenges will be crucial for developing targeted interventions that can enhance the successful adoption and sustainability of health information systems like DHIS2, ultimately contributing to improved health outcomes in the region.

## Data Availability

The raw data supporting the conclusions of this article will be made available by the authors, without undue reservation.
