# Supplementary material for: Unlocking health insights: exploring intention to adopt district health information systems in Bahir Dar City, northwest Ethiopia
Source: Front Digit Health. 2025 Feb 25;7:1449510. doi: 10.3389/fdgth.2025.1449510 (PMC11895800; doi:10.3389/fdgth.2025.1449510)
Supplement: Supplementary file 1 [file Datasheet1.pdf]

<u>Tool used to assess intention to use District health information system (DHIS2) in Bahir Dar City, Ethiopia.</u>

- In this study DHIS2= District health information system 2

Part One: This section aims to assess the socio-demographic factors of health professionals. Please encircle or fill in the blank space with the appropriate response that best represents your situation.

101. Sex of the respondent?	1. Male<br>2. Female
102. Age of the respondent?	____________________
103. What is your marital status?	1. Married 2. Single<br>3. Divorced 4. Widowed
104. Your educational status	1. Certificates<br>2. Diploma<br>3. Bachelor of Degree<br>4. Master's Degree and above
105. what is your field of specialization	1. Health Officer<br>2. Laboratory Technician<br>3. Environmental Health Technician<br>4. Pharmacist<br>5. Nurse<br>6. Medical doctor<br>7. Midwifery<br>8. Other ………………..
106. Experiences in years?	

Part two: This section is designed to assess the intention to use District Health Information System (DHIS2). Please indicate your level of agreement on each of the following statements using the Likert scale, where 1 represents "Strongly Disagree," 2 represents "Disagree," 3 represents "Neutral," 4 represents "Agree," and 5 represents "Strongly Agree." Please rate your level of agreement and encircle the corresponding number in front of each item.

I intend to use the DHIS2 system in the future	1	2	3	4	5

I will always try to use the DHIS2 system.	1	2	3	4	5
I plan to use the DHIS2 system in the future	1	2	3	4	5
**Part Three:** This section is designed to assess the attitude towards intention to use. Please rate your level of agreement on each of the following statements. The statements are expressed using the Likert scale, where 1 represents "Strongly Disagree," 2 represents "Disagree," 3 represents "Neutral," 4 represents "Agree," and 5 represents "Strongly Agree." Please indicate your level of agreement by selecting the appropriate number and encircling it in front of each item.					
Using DHIS2 is a good idea	1	2	3	4	5
Using DHIS2 is pleasant.	1	2	3	4	5
Using DHIS2 is beneficial.	1	2	3	4	5
I am interested to use DHIS2.	1	2	3	4	5
Overall, I like the idea of using DHIS2.	1	2	3	4	5
**Part Four:** This section is designed to assess perceived usefulness towards intention to use. Please rate your level of agreement on each of the following statements. The statements are expressed using the Likert scale, where 1 represents "Strongly Disagree," 2 represents "Disagree," 3 represents "Neutral," 4 represents "Agree," and 5 represents "Strongly Agree." Please indicate your level of agreement by selecting the appropriate number and encircling it in front of each item.					
Using the DHIS2 enhance my effectiveness in healthcare delivery.	1	2	3	4	5
Using the DHIS2 increase my productivity in my work.	1	2	3	4	5
Using the DHIS2 enable me to accomplish tasks more quickly.	1	2	3	4	5
I found using the DHIS2 useful.	1	2	3	4	5
**Part Five:** This section is designed to assess perceived ease of use towards intention to use. Please rate your level of agreement on each of the following statements. The statements are expressed using the Likert scale, where 1 represents "Strongly Disagree," 2 represents "Disagree," 3 represents "Neutral," 4 represents "Agree," and 5 represents "Strongly Agree." Please indicate your level of agreement by selecting the appropriate number and encircling it in front of each item.					
Learning to use the DHIS2 is easy for me.	1	2	3	4	5

DHIS2 system is clear and understandable to use.	1	2	3	4	5
It is easy for me to become skillful at using the DHIS2.	1	2	3	4	5
I found the DHIS2 to be flexible to interact with.	1	2	3	4	5
**Part Six:** This section is designed to assess computer skill of health professionals. Please indicate your level of agreement by selecting the appropriate number and encircling it in front of each item.					
Do you have basic level of word processing knowledge (e.g. MS Word)	1. Yes<br>2. No				
Do you have basic level of web surfing Skill with any Internet browse?	1. Yes<br>2. No				
Do you have basic level of computer spread sheet processing skill (MS Excel)?	1. Yes<br>2. No				
Do you have basic level of presentation software skill (e.g. MS PowerPoint)?	1. Yes<br>2. No				
Do you have basic level of emailing skill?	1. Yes<br>2. No				
**Part Seven:** This section is designed to assess organizational factors towards DHIS2. Please encircle or fill in the blank space with the appropriate response that best represents your situation.					
Have you experience in the use of DHIS2 software?	1. Yes<br>2. No				
Is there a specific person (group) available to assist DHIS2 difficulties' in your facility	1. Yes<br>2. No				
Specialized instruction and education concerning software about DHIS2 is available in your facility?	1. Yes<br>2. No				
Specialized programs and training about DHIS2 are available in your facility?	1. Yes<br>2. No				
Did you attend training on DHIS2?	1. Yes<br>2. No				If no, skip to Q710

If "Yes "For how many days training on DHIS2 were made?	1. 3-5 days<br>2. 6-7 days<br>3. 8-10 days<br>4. >10 days	
In which form was the training taken?	1. Only theoretical<br>2.Both theoretical & Practical	
How would you rate your level of training on DHIS2?	1. Very poor 2. Poor<br>3. Good 4. Very Good	
The training you received was adequate?	1. Strongly agree 2. Agree<br>3. Disagree<br>4. Strongly disagree	
Is there the necessary equipment for DHIS2 implementation in your facility?	1. Yes<br>2. No	If no, skip to Q712
If " yes ", which of the following equipment are assigned to DHIS2?	1. Power supply<br>2.Telephone<br>3.Computer hardware and software<br>4. Internet or any kind of network<br>5. printer<br>6. Hard copy manual	
Do you have internet access in your facilities?	1. Yes<br>2. No	
How do you mostly access the internet for your work? Through	1. Wi-Fi<br>2. Internet service provider network<br>3. Mobile provider's wireless modern<br>4. Other………………..	
Currently, how would you rate the internet access provided at your work of place?	1. Very satisfied<br>2. Satisfied<br>3. Unsatisfied<br>4. Very unsatisfied	
